# What Is the Nutritional Composition of Ultra-Processed Food Marketed in Italy?

**DOI:** 10.3390/nu13072364

**Published:** 2021-07-10

**Authors:** Giulia Lorenzoni, Rita Di Benedetto, Marco Silano, Dario Gregori

**Affiliations:** 1 Unit of Biostatistics, Epidemiology and Public Health, Department of Cardiac, Thoracic, Vascular Sciences and Public Health, University of Padova, 35131 Padova, Italy; giulia.lorenzoni@unipd.it; 2 Unit of Human Nutrition and Health, Department of Food Safety, Nutrition and Veterinary Public Health, Italian National Institute of Health, 00161 Roma, Italy; rita.dibenedetto@iss.it (R.D.B.); marco.silano@iss.it (M.S.)

**Keywords:** ultra-processed food, front-of-pack labeling, food classification

## Abstract

The present study aimed to provide a descriptive analysis of the nutrient profile of ultra-processed foods (UPFs) marketed in Italy according to three front-of-pack labeling (FOPL) schemes implemented by France, i.e., the Nutriscore; by the United Kingdom, i.e., Multiple Traffic Lights (MTL); and by Italy, i.e., the NutrInform battery. The analysis was made in fourteen food product categories, corresponding to 124 foods. The application of the Nutriscore scheme showed that a significant proportion of foods (23%) were awarded an A or B. Furthermore, the analysis according to the MTL showed that food products that were above the threshold (“red”) for fat, saturated fats, sugars, and salt ranged from 13% to 31%. Interestingly, even though all foods considered in the analysis were UPF, they were heterogeneous in nutritional composition, as demonstrated by the FOPL schemes applied, showing that UPF represent a heterogeneous group of foods with different characteristics. Such a finding may have relevant implications for epidemiological studies that analyze the association between UPF consumption and health outcomes, suggesting the need for better characterization of the effects of UPF intake on human health.

## 1. Introduction

A group of Brazilian researchers has proposed a new type of food classification, named NOVA [1]. This system is based on the intensity of food processing. It distinguishes between four food groups, i.e., unprocessed or minimally processed foods, processed culinary ingredients, processed foods, and ultra-processed foods (UPFs) [2].

Such a new approach represents a paradigm shift in thinking about food classification. For a long time, food classification has been mainly based on nutrient content. The literature aimed at identifying thresholds beyond which the consumption of certain foods or nutrients may represent a risk for human health, and these thresholds were translated into dietary recommendations in the context of public health initiatives. Conversely, the NOVA classification is based on the intensity of food processing, introducing the idea that the criteria to be taken into account in food classification are the processes the foods undergo before entering the market instead of the nutrient content.

In a short time, the proposed classification has become a popular tool in nutrition research, and it has been adopted for the assessment of the health impact of food in nutritional surveys. Studies show that the purchase and consumption of UPF has increased in the recent years [3,4], and UPF consumption has been shown to range from 30% to 50% of the daily energy intake, depending on the country [5,6]. Furthermore, given the relevant contribution of UPF to daily energy intake, it has been evaluated whether UPF intake is associated with adverse health outcomes. Interestingly, observational studies have suggested that the consumption of UPF is associated with a higher risk of cardiovascular diseases, metabolic diseases, cancer, and all-cause and cardiovascular mortality [7,8,9,10].

The suggested adverse health consequences of UPF consumption have also influenced public health nutrition policies. As a result, a growing interest in adopting such a new classification tool in formulating dietary recommendations and public health approaches to improve population diets has emerged.

Although the NOVA classification has relevant implications in nutrition research and public health nutrition, it has been shown that classifying food according to the criteria proposed by the NOVA classification is not straightforward, and recent studies have shown a poor agreement between different classification systems [11,12]. This is a relevant issue in nutrition research, because using different classifications may bias study results [13].

Furthermore, the classification system also affects the development and implementation of policy initiatives. A striking example of this fact is front-of-pack labeling (FOPL). FOPL has been advocated as one of the most valuable public health strategies to inform consumers and to influence their choices [14]. However, several FOPLs have been proposed [15], based on nutrient content but using different thresholds, e.g., the traffic light system, the Nutriscore, the NutrInform battery, the Health Star Rating (HSR). No international consensus has yet been reached on the FOPL to be used. After introducing the new classification system based on the extent of food processing, researchers have become interested in understanding the relationship between such an approach and the existing FOPLs [11]. Interestingly, a recent study has shown a low agreement between the NOVA classification and the HSR system adopted in Australia [11].

The present study aims to provide a descriptive analysis of the nutrient profile of UPFs marketed in Italy using three FOPL schemes, implemented by France, i.e., the Nutriscore; by the UK, i.e., Multiple Traffic Lights (MTL); and by Italy, i.e., the NutrInform battery.

## 2. Materials and Methods

A set of UPF product categories marketed in Italy was considered in the present study, i.e., cookies, biscuits, ready-to-eat cereals, savory biscuits, rusks, ice-creams, dehydrated meals, frozen meals, frozen meat/fish meals, refrigerated meals, frozen pizzas, snack cakes, croissants, and salty snacks.

For each food product category, the top ten selling products in 2018 were considered, according to the official data from the Unione Italiana Food. Food products that were no longer available in the Italian market in 2021 were excluded from the analysis.

Nutrition facts were taken from the official Italian food companies’ websites. If not reported on a website, nutrition facts reported on the product package were considered.

### 2.1. FOPL Schemes

Three FOPL schemes were employed to classify the UPFs, the Nutriscore [16], the NutrInform battery [17], and the MTL schemes. They were chosen because one is directive/graded, i.e., the Nutriscore, one is semi-directive/color-coded, i.e., the MTL, and the other is non-directive/monochromatic, i.e., the NutrInform Battery.

The Nutriscore, previously known as 5-Colour Nutrition Label, classifies food into five classes identified by both a color and a letter from A to E, with E being food with the worse nutrient profile. The classification is based on the United Kingdom Food Standards Agency’s nutrient profiling system. It was subsequently adapted to the French setting after the Nutriscore was proposed and adopted in France for the first time in 2017. In a short time, the use of the Nutriscore has been introduced/proposed by other European countries, including Belgium, Luxembourg, Spain, Germany, the Netherlands, and Switzerland. 

The score calculation is made according to an algorithm that considers energy, total fats, saturated fats, sugars, protein, fiber, and salt content. The reference base for the computation of the Nutriscore is 100 g for solids and 100 mL for liquids. The Nutriscore was calculated for each food product using a web-based resource [18].

The UK government proposed the MTL scheme in 2013. Similarly to the Nutriscore, the use of the FOPL scheme is voluntary. It is a color-coded system showing if a product is low (green), medium (amber), or high (red) in fat, saturated fats, sugars, and salts. The following classification criteria, based on 100 g, were employed:

- ≤3 g/100 g green, >3 g/100 g and ≤17.5 amber, >17.5 g/100 g for fat;
- ≤1.5 g/100 g green, >1.5 g/100 g and ≤5 amber, >5 g/100 g for saturates;
- ≤5 g/100 g green, >5 g/100 g and ≤22.5 amber, >22.5 g/100 g for sugars;
- ≤0.3 g/100 g green, >0.3 g/100 g and ≤1.5 amber, >1.5 g/100 g for salt.

The NutrInform battery is a FOPL scheme developed by Italy, and notified to the European Commission in January 2021. It is a non-directive, nutrient-specific label. This scheme is based on the “reference intakes” label, with an added battery symbol indicating the amount of energy and nutrients (total and saturated fats, sugars, and salt). The reference base for the NutrInform battery is a serving of the food product, and it is reported as the percentage of the daily intake. The NutrInform battery was implemented using a web-based resource [19].

### 2.2. Statistical Analysis

Continuous data were reported as I quartile/median/III quartile, while categorical data were reported as percentages (absolute numbers).

The Wilcoxon–Kruskal–Wallis test was performed to compare nutrient content distribution among the food product categories.

Analyses were performed using R software [20].

## 3. Results

Fourteen product categories were considered in the analysis, corresponding to 124 food products, since sixteen foods were not considered in the current analysis as they were no longer available in the Italian market. Examples of foods included in each category are provided in Table S1.

Cookies, crackers, and biscuits were the most energy-dense foods, with a median energy content of 1999 kJ, 1850 kJ, and 1817.5 kJ, respectively (Table 1). Ice-creams, cookies, snack cakes, and croissants were the highest in fats, saturated fats, and sugars. Foods with the highest fiber content were rusks, ready-to-eat cereals, and crackers with a median fiber content of 6.05 g/100 g, 5.8 g/100 g, and 5.4 g/100 g, respectively. Crackers were also the highest in salt content, i.e., median of 1.975 g/100 g, followed by frozen rusks, i.e., 1.357 g/100 g.

### FOPL Schemes Application

The application of the Nutriscore scheme showed that 11% of food products were labeled E, where croissants (44%), snack cakes (40%), and ice-creams (30%) were the categories most represented. (Table 2). Overall, the most represented Nutriscore categories were D and C, with 33% of foods each. Interestingly, 23% of foods were awarded an A or B by the Nutriscore.

Concerning the MTL, less than one-third of the food products overall were above the thresholds (“red”) for fat (23%), saturated fats (31%), sugars (23%), and salt (13%). On the other hand, foods were most often classified as medium (“amber”) for the content of total fats (65%), saturated fats (29%), sugars (31%), and salt (74%). When considered all together, no foods were below the threshold for green in all nutrient categories. Eight foods (five dehydrated meals, two refrigerated meals, and one frozen meal) were green in total fats, saturated fats, and sugars, but they were all amber concerning the salt content, except one, which was red. On the other hand, no foods received four reds.

Finally, the NutrInform battery was applied to two products for each category, one was awarded an A and one was awarded an E by the Nutriscore (Figure S1).

## 4. Discussion

The present study analyzed the nutritional composition of UPF marketed in Italy using three FOPL schemes, the Nutriscore, the NutrInform battery, and the MTL.

According to the MTL, a significant proportion of the foods analyzed was green in fat, saturated fat, sugars, or salt content and were awarded an A or B by the Nutriscore (23% of foods). Such a finding deserves careful consideration since it is noteworthy that the NOVA classification has come with the recommendation of limiting the consumption of UPF, but in the present study, a non-negligible proportion of foods was classified as being of a balanced nutritional composition by the three FOPL schemes, used inside and outside the European Union. Similarly, a recent study conducted in Honduras showed that the proportion of processed and ultra-processed food marketed in the country that met criteria for sugar content ranged from 25% to 60%, depending on the classification system used [21]. The proportion of processed and ultra-processed foods that met the criteria ranged from 32.6% to 63.6% for saturated fats.

It is noteworthy that the idea behind the NOVA classification is that industrial food processing itself is harmful to human health [22]; however, UPF is a category that includes several types of food products that undergo heterogeneous food processing techniques. For this reason, the fact that some recent studies concluded that a certain proportion of daily UPF consumption is associated with a higher risk of non-communicable diseases NCDs and death [8,23,24] is uninformative, because this information does not take into account the type of UPF and the type of processing they underwent. Therefore, it would be better to analyze the contribution of each kind of UPF to better characterize the UPF hazard to human health. Conversely, we cannot rule out that the observed association between UPF consumption and adverse health outcomes hides the well-known association between the consumption of foods of poor nutritional composition and adverse health outcomes. As an example, Bonaccio et al. [8] found that daily proportion of UPF was associated with cardiovascular death. Nevertheless, pastries/cakes/puddings were in the top three of foods contributing to the daily proportion of UPF intake, and, together with processed meat and pizza, accounted for 50% of daily UPF intake. The fact that UPF represent a heterogeneous group of foods with different characteristics is clearly shown in the present work. Even though all foods considered were UPFs, they were heterogeneous in the processing techniques employed and nutritional composition, as demonstrated by the FOPL applied.

Therefore, it is important to consider the culture and traditional context in which a food classification system has been developed. The adoption of one food classification system over another is a matter concerning governments worldwide. This issue stems from the idea that a food classification system may fit the dietary habits/food choices in the country where it has been developed very well. Conversely, the same classification system may not be adequate in another cultural context presenting different dietary habits. Moreover, regarding the FOPL, the Italian government has implemented the NutrInform battery that informs consumers, instead of giving them strict directions on consumption. For heterogenous food groups, such as UPF, the NutrInform battery, being nutrient specific and not directive, is the FOPL scheme that gives complete information on the nutritional composition of foods, allowing for informed choices.

Regarding study limitations, the main one is that not all UPF categories were considered in the analysis since data were not available about the top selling product for all categories. However, the food categories investigated provide an exhaustive representation of the UPFs sold in Italy. Another limitation of the study was that nine out of ten frozen meat/fish meals were pre-fried, and the Nutriscore recommends considering the nutritional facts after frying. However, all the products considered could be cooked in the oven as an alternative to frying.

## 5. Conclusions

The present descriptive analysis shows that the NOVA classification does not meet the nutritional composition criteria of the food products, as demonstrated by applying three widely used FOPL schemes. Present findings raise some points for future reference, including the improvement of UPF characterization in epidemiological studies that analyze the association between UPF intake and adverse health outcomes and the analysis of the validity of the NOVA classification.

## Figures and Tables

**Table 1:** Distribution of nutritional facts according to food category. Data are I quartile/median/III quartile. *p*-value of the Wilcoxon–Kruskal–Wallis test for the difference of the distribution of nutritional facts among food categories was <0.001 for all food categories.

	**Energy (kJ)**	**Total Fat (g/100 g)**	**Saturated Fat (g/100 g)**	**Total Carbohydrate (g/100 g)**
Cookies	1964.25/1999.00/2022.75	18.775/20.000/20.375	4.000/5.200/7.400	65.175/65.900/68.150
Biscuits	1808.50/1817.50/1840.25	9.400/10.500/12.000	1.100/1.450/3.250	69.925/75.300/76.375
Ready-to-eat cereals	1591.00/1604.00/1767.00	1.700/2.500/13.000	0.600/0.600/3.800	71.000/74.800/75.800
Crackers	1782.00/1850.50/1869.00	10.450/13.500/14.000	1.225/1.600/2.600	61.500/66.100/67.550
Rusks	1654.25/1675.00/1702.25	5.550/5.800/6.150	0.625/0.750/1.050	68.150/71.300/73.350
Ice-creams	1195.00/1270.00/1308.75	14.250/16.500/19.000	8.725/12.000/13.000	29.250/31.500/37.750
Snack cakes	1575.00/1745.50/1868.75	14.450/20.150/21.525	4.825/7.500/9.450	47.000/52.750/56.975
Croissants	1724.00/1732.00/1744.00	19.000/21.000/22.500	9.400/10.000/12.500	47.000/48.100/49.200
Dehydrated meals	473.00/489.50/750.50	1.000/1.650/2.000	0.100/0.450/0.575	23.250/24.500/34.600
Refrigerated meals	288.00/718.00/752.00	2.300/6.800/7.000	0.360/0.900/0.940	8.800/17.000/17.000
Frozen meals	512.00/679.00/746.00	4.500/8.200/9.700	1.600/2.300/2.900	6.200/12.800/13.400
Frozen pizzas	954.00/968.00/1105.00	8.000/8.900/9.800	3.100/3.600/4.300	25.000/30.000/33.000
Frozen meat/fish meals	805.75/887.00/979.00	7.625/8.950/10.025	0.625/1.100/2.025	12.450/21.250/22.750
Salty snacks	846.00/997.00/1161.00	7.200/10.200/16.100	0.900/2.800/6.750	23.000/25.000/25.500
	**Sugars (g/100 g)**	**Dietray Fiber (g/100 g)**	**Protein (g/100 g)**	**Salt (g/100 g)**
Cookies	20.000/21.250/23.375	3.000/3.250/3.800	6.450/6.850/7.075	0.534/0.650/0.788
Biscuits	18.850/19.500/22.250	2.625/3.600/7.300	8.500/8.550/9.300	0.728/0.750/0.958
Ready-to-eat cereals	12.000/24.100/26.000	4.300/5.800/7.600	7.000/8.300/8.500	0.630/0.900/0.940
Crackers	2.125/2.500/3.000	4.800/5.400/6.325	10.000/10.000/10.750	1.713/1.975/2.238
Rusks	5.250/6.050/6.725	4.650/6.050/7.425	11.025/11.450/12.225	1.062/1.357/1.500
Ice-creams	22.250/23.000/27.000	0.725/1.350/1.975	3.525/4.150/4.550	0.130/0.150/0.238
Snack cakes	25.075/29.150/35.375	3.000/3.900/3.900	6.000/6.750/9.125	0.265/0.340/0.500
Croissants	16.000/20.000/21.000	1.600/1.700/1.900	6.400/7.000/7.300	0.460/0.500/0.600
Dehydrated meals	0.400/0.700/0.775	0.500/0.500/1.400	2.200/2.550/3.350	0.783/0.810/1.108
Refrigerated meals	2.000/2.500/2.600	1.500/2.300/2.900	2.200/5.400/6.100	0.520/0.660/0.800
Frozen meals	0.700/1.700/2.400	0.800/1.100/1.600	5.600/8.700/10.300	0.700/0.800/1.000
Frozen pizzas	2.200/2.900/3.200	1.875/2.150/2.450	9.500/10.000/10.100	1.100/1.300/1.300
Frozen meat/fish meals	0.550/0.850/1.100	0.800/1.000/1.100	9.625/12.000/14.000	0.853/0.970/1.225
Salty snacks	1.350/2.400/2.900	1.050/1.400/1.500	3.800/6.000/9.650	0.950/1.200/1.300

**Table 2:** Classification of foods according to the Nutriscore and the MTL schemes. The Nutriscore classifies food into five classes identified by both a color and a letter from A to E, with E being food with the worse nutrient profile. Data are percentages (absolute number).

	**Cookies**	**Biscuits**	**Ready-to-Eat Cereals**	**Crackers**	**Rusks**
Nutriscore					
A	0% (0)	0% (0)	11% (1)	0% (0)	10% (1)
B	0% (0)	0% (0)	33% (3)	0% (0)	20% (2)
C	10% (1)	50% (5)	22% (2)	70% (7)	70% (7)
D	80% (8)	50% (5)	33% (3)	10% (1)	0% (0)
E	10% (1)	0% (0)	0% (0)	20% (2)	0% (0)
Multiple Traffic light					
Fat: Amber	20% (2)	100% (10)	33% (3)	100% (10)	100% (10)
Green	0% (0)	0% (0)	56% (5)	0% (0)	0% (0)
Red	80% (8)	0% (0)	11% (1)	0% (0)	0% (0)
Saturates: Amber	40% (4)	20% (2)	11% (1)	50% (5)	0% (0)
Green	10% (1)	60% (6)	67% (6)	30% (3)	100% (10)
Red	50% (5)	20% (2)	22% (2)	20% (2)	0% (0)
Sugars: Amber	60% (6)	60% (6)	44% (4)	20% (2)	70% (7)
Green	0% (0)	10% (1)	0% (0)	80% (8)	30% (3)
Red	40% (4)	30% (3)	56% (5)	0% (0)	0% (0)
Salt: Amber	100% (10)	100% (10)	100% (9)	0% (0)	80% (8)
Green	0% (0)	0% (0)	0% (0)	0% (0)	0% (0)
Red	0% (0)	0% (0)	0% (0)	100% (10)	20% (2)
	**Ice-creams**	**Snack Cakes**	**Croissants**	**Dehydrated Meals**	**Refrigerated Meals**
Nutriscore					
A	0% (0)	0% (0)	0% (0)	0% (0)	20% (1)
B	0% (0)	0% (0)	0% (0)	0% (0)	80% (4)
C	10% (1)	10% (1)	0% (0)	83% (5)	0% (0)
D	60% (6)	50% (5)	56% (5)	17% (1)	0% (0)
E	30% (3)	40% (4)	44% (4)	0% (0)	0% (0)
Multiple Traffic Light					
Fat: Amber	50% (5)	40% (4)	11% (1)	17% (1)	60% (3)
Green	10% (1)	0% (0)	0% (0)	83% (5)	40% (2)
Red	40% (4)	60% (6)	89% (8)	0% (0)	0% (0)
Saturates: Amber	0% (0)	30% (3)	0% (0)	0% (0)	20% (1)
Green	10% (1)	0% (0)	0% (0)	100% (6)	80% (4)
Red	90% (9)	70% (7)	100% (9)	0% (0)	0% (0)
Sugars: Amber	30% (3)	10% (1)	89% (8)	0% (0)	0% (0)
Green	0% (0)	0% (0)	0% (0)	100% (6)	100% (5)
Red	70% (7)	90% (9)	11% (1)	0% (0)	0% (0)
Salt: Amber	0% (0)	60% (6)	100% (9)	83% (5)	100% (5)
Green	100% (10)	40% (4)	0% (0)	0% (0)	0% (0)
Red	0% (0)	0% (0)	0% (0)	17% (1)	0% (0)
	**Frozen Meals**	**Frozen Pizzas**	**Frozen Meat/Fish Meals**	**Salty Snacks**	**Overall (*n* = 124)**
Nutriscore					
A	44% (4)	0% (0)	20% (2)	14% (1)	8% (10)
B	22% (2)	22% (2)	40% (4)	14% (1)	15% (18)
C	22% (2)	56% (5)	30% (3)	29% (2)	33% (41)
D	11% (1)	22% (2)	10% (1)	43% (3)	33% (41)
E	0% (0)	0% (0)	0% (0)	0% (0)	11% (14)
Multipe Traffic light					
Fat: Amber	89% (8)	100% (9)	100% (10)	71% (5)	65% (81)
Green	11% (1)	0% (0)	0% (0)	0% (0)	11% (14)
Red	0% (0)	0% (0)	0% (0)	29% (2)	23% (29)
Saturates: Amber	78% (7)	89% (8)	30% (3)	29% (2)	29% (36)
Green	22% (2)	0% (0)	70% (7)	43% (3)	40% (49)
Red	0% (0)	11% (1)	0% (0)	29% (2)	31% (39)
Sugars: Amber	0% (0)	0% (0)	0% (0)	14% (1)	31% (38)
Green	100% (9)	100% (9)	100% (10)	86% (6)	46% (57)
Red	0% (0)	0% (0)	0% (0)	0% (0)	23% (29)
Salt: Amber	78% (7)	100% (9)	90% (9)	71% (5)	74% (92)
Green	11% (1)	0% (0)	0% (0)	14% (1)	13% (16)
Red	11% (1)	0% (0)	10% (1)	14% (1)	13% (16)

The names of the food products’ categories are in bold.

## Data Availability

The data presented in this study are available on request from the corresponding author.

## Supplementary Materials

The following are available online at https://www.mdpi.com/article/10.3390/nu13072364/s1. Table S1: Food categories’ examples, Figure S1: NutrInform Battery calculated on foods categorized as A or E by the Nutriscore for each food category. When not reported, no foods were categorized as A or E in that category. Not Available (NA) means that the per serving information was not available nor on the website nor on the product package.
