# Supplementary material for: What Is the Nutritional Composition of Ultra-Processed Food Marketed in Italy?
Source: Nutrients. 2021 Jul 10;13(7):2364. doi: 10.3390/nu13072364 (PMC8308580; doi:10.3390/nu13072364)
Supplement: Supplementary file 1 [file nutrients-13-02364-s001.zip › nutrients-1275635-supplementary.pdf]

Table S1. Food categories’ examples

**Food categories**	
Cookies	Shortbread cookies that may be added with other ingredients, e.g., chocolate, chocolate chips, frosting
Biscuits	Dry plain biscuits
Croissants	Individually wrapped plain or filled croissants
Snack cakes	Individually wrapped snack cakes
Rusks	Toasted and crisp slices of bread to spread usually eaten for breakfast
Crackers	Crackers
Ready-to-eat cereals	Breakfast cereals mainly made from wheat, oats, rice, and maize added with flavors
Dehydrated meals	Pre-cooked dried meals that need to be rehydrated with boiled water in a pot for a few minutes, e.g., dehydrated risotto and soup
Refrigerated meals	Refrigerated ready meals, e.g., vegetables soup, vegetables couscous
Frozen pizzas	Frozen pizzas, e.g., ham and cheese pizza, ham and mushrooms pizza, vegetables pizza, “Margherita” pizza
Frozen meals	Frozen ready meals, e.g., pasta and sauce, paella, filled pasta
Frozen meat/fish meals	Frozen meat/fish recipes, e.g., fish balls, chicken nuggets, crumbed fish fillets
Salty snacks	Salty snacks, e.g., mini pizzas, onion rings, spring rolls
Ice-creams	Individually wrapped ice creams, e.g., cones, bars, cups

Figure S1. NutrInform Battery calculated on foods categorized as A or E by the Nutriscore for each food category. When not reported, no foods were categorized as A or E in that category. Not Available (NA) means that the per serving information was not available nor on the website nor on the product package.

	A	E
Biscuits	-	-
Cookies	-	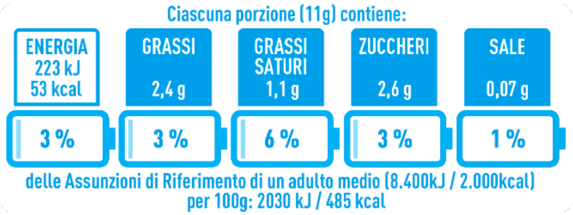
Croissants	-	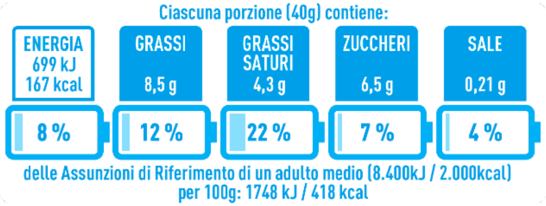
Snack cakes	-	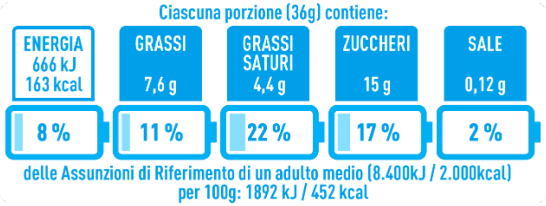
Rusks	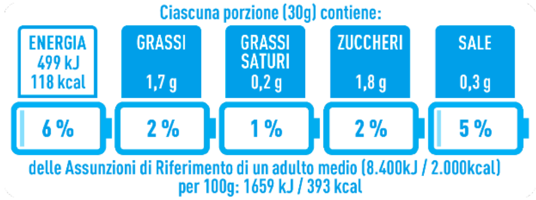	-

Salty biscuits	-	Ciascuna porzione (29g) contiene:<br>ENERGIA 546 kJ 130 kcal 7 % | GRASSI 4,2 g 6 % | GRASSI SATURI 2,5 g 13 % | ZUCCHERI 0,6 g 1 % | SALE 0,64 g 11 %<br>delle Assunzioni di Riferimento di un adulto medio (8.400kJ / 2.000kcal)<br>per 100g: 1881 kJ / 447 kcal
Ready-to-eat cereals	Ciascuna porzione (30g) contiene:<br>ENERGIA 467 kJ 110 kcal 6 % | GRASSI <0,5 g 1 % | GRASSI SATURI 0,2 g 1 % | ZUCCHERI 3,2 g 4 % | SALE 0,27 g 5 %<br>delle Assunzioni di Riferimento di un adulto medio (8.400kJ / 2.000kcal)<br>per 100g: 1557 kJ / 368 kcal	-
Dehydrated meals	-	-
Refrigerated meals	Ciascuna porzione (110g) contiene:<br>ENERGIA 790 kJ 189 kcal 9 % | GRASSI 7,7 g 11 % | GRASSI SATURI 1 g 5 % | ZUCCHERI 2,2 g 2 % | SALE 0,73 g 12 %<br>delle Assunzioni di Riferimento di un adulto medio (8.400kJ / 2.000kcal)<br>per 100g: 718 kJ / 172 kcal	-
Frozen pizzas	-	-
Frozen meals	Ciascuna porzione (250g) contiene:<br>ENERGIA 1282 kJ 304 kcal 15 % | GRASSI 8,8 g 13 % | GRASSI SATURI 1 g 5 % | ZUCCHERI 1,5 g 2 % | SALE 1,8 g 30 %<br>delle Assunzioni di Riferimento di un adulto medio (8.400kJ / 2.000kcal)<br>per 100g: 512 kJ / 122 kcal	-
Frozen meat/fish meals	NA	-
Salty snacks	NA	-
Ice-creams	-	Ciascuna porzione (86g) contiene:<br>ENERGIA 1171 kJ 280 kcal 14 % | GRASSI 17 g 24 % | GRASSI SATURI 11 g 55 % | ZUCCHERI 24 g 27 % | SALE 0,12 g 2 %<br>delle Assunzioni di Riferimento di un adulto medio (8.400kJ / 2.000kcal)<br>per 100g: 1362 kJ / 325 kcal

Ciascuna porzione”: each serving; “Energia”: energy; “Grassi”: fats; “Grassi saturi”; saturated fats; “Zuccheri”: sugars; “Sale”: salt; “delle assunzioni di riferimento di un adulto medio”: of reference intake for an adult
